# Supplementary material for: The Role of Mineral and Trace Element Supplementation in Exercise and Athletic Performance: A Systematic Review
Source: Nutrients. 2019 Mar 24;11(3):696. doi: 10.3390/nu11030696 (PMC6471179; doi:10.3390/nu11030696)
Supplement: Supplementary file 1 [file nutrients-11-00696-s001.pdf]

Supplementary materials

Table S1 *Study characteristics and EPHPP global rating*

	Author (year)	Study design	Participants		Outcome results of supplementation	EPHPP Global rating
			Group	Sample size (n=F)		
**Iron**						
	Dressendorfer, *et al.*, 1991 [1]	CT	Elite athletes	15(0)	Fe had no effect on exercise associated reduction in biomarkers of low Fe during a 20 day 500km running road race.	Moderate
	Govus, *et al.*, 2015 [2]	CT	Elite athletes	178(80)	HBmass increased in those with low and mid-baseline ferritin levels supplemented with Fe with no change in non-supplemented athletes.	Moderate
	Villanueva, *et al.*, 2011 [3]	RCT	Elite athletes	35(0)	Fe increased iron, ferritin, transferrin saturation, haematocrit, mean corpuscular volume greater than placebo.	Moderate
	Flynn, *et al.*, 2003 [4]	RCT	Athletes	22(0)	Fe increase ferritin but had no effect on serum Fe, haematocrit or markers of immune function following 2 weeks of intensive training or in the following 8 week recovery period.	Moderate
	Friedmann, *et al.*, 1999 [5]	RCT	Elite athletes	17(0)	Fe had no effect on physiological parameters measured at moderate altitude (1800m) over 18 days.	Moderate
	Fogelholm, *et al.*, 1992 [6]	RCT	Athletes	33(33)	Ferritin and haemoglobin levels improved but no improvement in aerobic performance or lactate concentrations with Fe supplementation, compared to placebo.	Moderate
	Klingshirn, *et al.*, 1992 [7]	RCT	Athletes	18(18)	Ferritin and iron binding capacity levels improved but there was no improvement in aerobic performance or lactate concentrations with Fe supplementation, compared to placebo.	Strong
	Hegenauer, *et al.*, 1983 [8]	CT	Non-athletes	40(40)	Fe had no effect on ferritin, haemoglobin or haematocrit.	Moderate
	Jensen, *et al.*, 1991 [9]	RCT	Non-athletes	13(13)	Ferritin levels improved with Fe supplementation but no association with VO_{2peak}.	Moderate
	Hinton and Sinclair, 2007 [10]	RCT	Non-athletes	20(17)	Fe failed to change VO_{2peak}, but improved ferritin and energetic efficiency during submaximal exercise and prevented the decline in ventilatory threshold observed in the placebo group, with the greatest change evident in those with the lowest ferritin levels.	Strong
	Magazanik, *et al.*, 1991 [11]	RCT	Non-athletes	28(28)	Fe supplementation maintained ferritin levels and increased VO_{2peak} at 3 but not 6 weeks.	Moderate

Author (year)	Study design	Participants		Outcome results of supplementation	EPHPP Global rating
		Group	Sample size (n=F)		
LaManca and Haymes, 1993 [12]	RCT	Non-athletes	20(20)	Fe increased ferritin, Hb and VO_{2peak} and lowered lactate concentrations following submaximal exercise compared to placebo.	Moderate
Hinton, *et al.*, 2000 [13]	RCT	Non-athletes	42(42)	15km time trial improved following high intensity endurance training with Fe supplementation and was accompanied by increases in serum ferritin and Hb.	Strong
McClung, *et al.*, 2009 [14]	RCT	Military recruits	171(171)	Fe attenuated blood markers of exercise-induced deficiency, but the supplemented Fe-deficient anaemia group completed a two mile time trial 110s faster.	Moderate
DellaValle and Haas, 2014 [15]	RCT	Athletes	48(48)	Fe showed a slower rate of lactate accumulation at the 1km and 2 km stages of a 4 km time trial and these athletes recovered quicker post-exercise. The lowest baseline Fe, supplemented with Fe improved 4 km time trial energy efficiency greater than placebo.	Strong
Yoshida, *et al.*, 1990 [16]	RCT	Athletes	20(20)	Fe improved ferritin, running velocity both at lactate threshold and onset of blood lactate accumulation and 3000m running performance.	Moderate
Ohira, *et al.*, 1979 [17]	CT	Non-athletes	20(14)	Fe supplementation improved VO_2 kinetics and heart rate compared to placebo.	Moderate
Garvican, *et al.*, 2014 [18]	Intervention	Elite athletes	27(14)	Six weeks of intravenous (IV) Fe was superior to high dose oral supplementation for improving VO_{2max}, max running time, with trivial effects on maximal blood lactate.	Moderate
Peeling, *et al.*, 2007 [19]	RCT	Athletes	20(20)	No performance effect of IV Fe was identified for VO_2 kinetics, heart rate or blood lactate compared to saline.	Moderate
Woods, *et al.*, 2014 [20]	RCT	Elite athletes	14(8)	Six weeks of IV Fe improved feelings of fatigue and Mood. Further, there was a small but potential important improvement in average 10-by-400m time during training compared to saline.	Moderate
Brutsaert, *et al.*, 2003 [21]	RCT	Non-athletes	20(20)	Fe increased post-fatiguing strength and showed a significant fatigue resistance in quadriceps, compared to placebo.	Strong
Mielgo-Ayuso, *et al.*, 2015 [22]	Randomized intervention	Elite athletes	20(20)	Fe for 11 weeks, enhanced markers of dynamic strength, the clean and jerk, power clean and total mean strength performance (ranging between ~10-40%) compared to controls.	Moderate

	Author (year)	Study design	Participants		Outcome results of supplementation	EPHPP Global rating
			Group	Sample size (n=F)		
	Burden, *et al.*, 2015 [23]	RCT	Elite athletes	15(9)	Ferritin, iron, transferrin saturation and hepcidin were improved in the iron group after supplementation and compared to placebo.	Strong
	Ishibashi, *et al.*, 2017 [24]	RCT	Athletes	14(0)	Fe increased hepcidin level greater than placebo.	Moderate
	Blee, *et al.*, 1999 [25]	CT	Elite athletes	15(NS)	Following Fe supplementation, ferritin increased compared to placebo. No difference in vertical jump test, 10s cycle power and 5x6s repeat sprint test and a 20m multi-stage shuttle run between the two groups.	Moderate
	Powell and Tucker, 1991 [26]	CCT	Athletes	10(10)	There was no difference between placebo or Fe in blood markers of iron metabolism or VO_2 kinetics.	Moderate
	Ashenden, *et al.*, 1999 [27]	Retrospective	Elite athletes	134(73)	Fe supplementation increased corpuscular haemoglobin, mean corpuscular volume and decreased reticulocyte volume distribution and cell haemoglobin concentration.	Weak
	Newhouse, *et al.*, 1989 [28]	RCT	Athletes	40(40)	Fe increased ferritin levels but had no effect on VO_2 kinetics.	Moderate
	Telford, *et al.*, 1992 [29]	CT	Athletes	31(18)	Fe increased ferritin levels but had no effect on peak anaerobic power performance.	Weak
**Calcium**						
	Martin, *et al.*, 2007 [30]	RCCT	Non-athletes	26(26)	Ca effectively attenuated the exercise-induced loss of Ca and altered the usual Ca loss associated with exercise into positive retention.	Moderate
	Zorbas, *et al.*, 2000 [31]	CT	Athletes	40(0)	Ca did not affect Ca balance or VO_{2peak} during normal endurance training or restricted training for 12 months. iPTH was decreased with restricted activity, regardless of additional Ca and the supplemented-restricted activity group decrease iPTH much greater that non-supplemented.	Moderate
	Zorbas, *et al.*, 2000 [32]	CT	Athletes	40(0)	Ca deficient athletes undergoing training restriction had lower PTH over time (12 months) but with no effect on VO_2 kinetics.	Moderate
	Kohrt, *et al.*, 2018 [33]	Interpreted time series	Non-athletes	11(0)	Ca attenuated the usual exercise-induced increase PTH, compared with saline infusion.	Moderate
	Guillemant, *et al.*, 2004 [34]	Cohort	Athletes	12(0)	Ca attenuated the usual exercise-induced increase in PTH.	Moderate
	Haakonssen, *et al.*, 2015 [35]	RCT	Athletes & Elite athletes	32(32)	Ca decreased with the onset of exercise but with Ca supplementation and attenuated the ca loss before and continued for > 90 mins following the cessation	Moderate

	Author (year)	Study design	Participants		Outcome results of supplementation	EPHPP Global rating
			Group	Sample size (n=F)		
					of exercise. Further, PTH was lower than controls before, throughout exercise and during recovery.	
	Cinar, *et al.*, 2009 [36]	Cohort	Athletes	30(0)	Ca had no effect on potassium or magnesium, with or without exhaustive exercise.	Weak
	Cinar, *et al.*, 2009 [37]	Cohort	Athletes	30(0)	Ca had no effect on testosterone, with or without exhaustive exercise.	Weak
	Cinar, *et al.*, 2009 [38]	Cohort	Athletes	30(0)	Ca had no effect on adrenocorticotropic hormone and cortisol levels with or without exhaustive exercise.	Weak
	Cinar, *et al.*, 2010 [39]	Cohort	Athletes	30(0)	Ca had no effect on leukocyte count, erythrocyte count, haemoglobin, haematocrit, and thrombocyte with or without exhaustive exercise.	Weak
	Cinar, *et al.*, 2010 [40]	Cohort	Athletes	30(0)	Ca had no effect on Glucose or Insulin Levels with or without exhaustive exercise.	Weak
**Magnesium**						
	Cordova Martinez, *et al.*, 2017 [41]	CT	Elite athletes	12(0)	Mg supplementation maintained serum Mg through the competitive basketball season with no change in other blood parameters.	Moderate
	Molina-Lopez, *et al.*, 2012 [42]	Interpreted time series	Elite athletes	12(0)	Mg supplementation maintained serum Mg through the competitive handball season.	Moderate
	Setaro, *et al.*, 2014 [43]	RCT	Elite athletes	25(0)	Mg reduced lactate production and increased countermovement jump performance, with no change in the control group.	Moderate
	Brilla and Haley, 1992 [44]	CT	Non-athletes	26(NS)	Mg induced an increase in both absolute and relative strength, compared to placebo.	Moderate
	Dmitrasinovic, *et al.*, 2016 [45]	RCT	Athletes	23(0)	Mg supplementation resulted in an increase of adrenocorticotropic hormone, while reducing cortisol concentrations, abolished the exercise-induced increase in IL-6 level and neutrophil/lymphocyte ratio, compared to controls.	Moderate
	Finstad, *et al.*, 2001 [46]	RCT	Non-athletes	32(32)	Mg supplementation increased circulating Mg but had no effect on blood pressure, anaerobic treadmill or incremental (aerobic) treadmill performance	Strong
	Kass, *et al.*, 2013 [47]	RCT	Non-athletes	16(0)	Mg improved post exercise blood pressure but had no effect on maximal 30 minute cycle or 3x 5 second isometric bench press.	Weak
	Zorbas, *et al.*, 1999 [48]	CT	Athletes	40(0)	Mg supplementation did not affect the negative Mg balance induced by 12 months of reduced activity.	Moderate
	Zorbas, *et al.*, 1998 [49]	CT	Athletes	40(0)	Mg supplementation did not affect the negative Mg balance induced by 12 months of reduced activity	Moderate

Author (year)	Study design	Participants		Outcome results of supplementation	EPHPP Global rating
		Group	Sample size (n=F)		
				and had no effect on maintaining VO_{2peak} throughout the restricted period.	
Porta*, et al.*, 2013 [50]	Cohort	Military recruits	15(NS)	Mg resulted in a reversal of increased Mg from pre-to-post exercise during a standardized military 2400m run.	Weak
Terblanche*, et al.*, 1992 [51]	RCT	Athletes	20(4)	Mg supplementation had no effect on marathon running performance.	Moderate
Weller*, et al.*, 1998 [52]	RCT	Athletes	20(4)	Mg supplement showed no difference in aerobic capacity, neuromuscular function or exercise haematological parameters, compared to placebo. Although muscle Mg correlated with total Mg concentration in mononuclear leukocytes, with an inverse correlation between muscle and red cell Mg.	Moderate
Petrovic*, et al.*, 2016 [53]	RCT	Athletes	23(0)	Mg mitigated exercise-induced DNA damage.	Weak
Cinar*, et al.*, 2007 [54]	Cohort	Athletes	30(0)	Mg increased magnesium, copper, and zinc levels with and without exhaustive exercise.	Weak
Cinar*, et al.*, 2008 [55]	Cohort	Athletes	30(0)	Mg had no effect on adrenocorticotropic hormone and cortisol levels with or without exhaustive exercise.	Weak
Cinar*, et al.*, 2007 [56]	Cohort	Athletes	30(0)	Mg had no effect on erythrocyte, haemoglobin, thrombocyte or leukocyte count.	Weak
Cinar*, et al.*, 2011 [57]	Cohort	Athletes	30(0)	Mg had no effect on testosterone, with or without exhaustive exercise.	Weak
Cinar*, et al.*, 2008 [58]	Cohort	Athletes	30(0)	Mg had no effect on Glucose or Insulin Levels with or without exhaustive exercise.	Weak
Kass and Poeira, 2015 [59]	Cohort	Athletes	13(6)	Acute Mg supplementation increased 1RM compared to no change in in the chronic Mg condition. Further, acute Mg maintained these gains the following day, whereas the control group decreased.	Weak
Veronese*, et al.*, 2014 [60]	RCT	Non-athletes	139(NS)	Mg had no effect on neuromuscular or handgrip strength, but did improve standard markers of physical function and was more pronounced in those with lower baseline Mg.	Strong
Gulick*, et al.*, 2012 [61]	RCT	Non-athletes	38(26)	Acute topical Mg application had no effect on either dorsiflexion ROM or incremental cycling exercise time trial.	Moderate
Mooren*, et al.*, 2003 [62]	RCT	Non-athletes	20(0)	Mg supplementation had no effect on human granulocyte signalling and function following a bout of exhaustive exercise.	Weak

	Author (year)	Study design	Participants		Outcome results of supplementation	EPHPP Global rating
			Group	Sample size (n=F)		
**Phosphate**						
	Buck, *et al.*, 2015 [63]	RCT	Athletes	12(12)	SP improved the first 20m, the fastest 20m and total sprint times (60m) compared to placebo or caffeine supplementation.	Weak
	Buck, *et al.*, 2015 [64]	RCT	Athletes	13(13)	Total sprint times were faster for each repeated sprint set faster overall supplemented with SP compared to placebo and beetroot juice.	Moderate
	Kopec, *et al.*, 2016 [65]	RCT	Athletes	11(0)	SP resulted in the fastest times for all sprints, compared to placebo or caffeine.	Moderate
	Brewer, *et al.*, 2015 [66]	RCT	Athletes	21(0)	SP improved power output during 4 days of intense race simulation in trained cyclists, with no change in placebo.	Moderate
	Folland, *et al.*, 2008 [67]	RCCT	Athletes	6(0)	SP improved power output and finishing time during a 1 km time trial, compared to placebo.	Moderate
	Brewer, *et al.*, 2013 [68]	RCCT	Athletes	12(0)	SP loading significantly improved VO_{2peak} compared to placebo. A follow up loading phase resulted in greater improvements.	Moderate
	Goss, *et al.*, 2001 [69]	RCCT	Athletes	12(0)	SP supplementation mitigate the rate of persevered exertion (RPE) during the mid-stages (of maximal treadmill running, but no change in physiological parameters were identified.	Moderate
	Kreider, *et al.*, 1992 [70]	RCT	Athletes	6(0)	SP improved VO_{2peak} in endurance athletes, increased blood haematocrit levels, glucose and other markers of metabolic demand. Further, SP enhanced ejection fraction and fractional shortening.	Moderate
	Czuba, *et al.*, 2009 [71]	RCT	Elite athletes	19(NS)	SP loading increased VO_{2peak}, reduced resting HR, max HR and HR at lactate threshold, all of which were maintained following lower dosing, compared to no change in placebo.	Moderate
	Czuba, *et al.*, 2008 [72]	RCT	Elite athletes	19(NS)	SP loading increased VO_{2peak}, reduced resting HR , max HR, compared to no change in placebo.	Moderate
	Cade, *et al.*, 1984 [73]	CT	Athletes	10(0)	SP increased 2,3-diphosphoglycerate (2,3-DPG) concentration, maximal oxygen uptake and blood lactate was attenuated, compared to placebo.	Moderate
	Stewart, *et al.*, 1990 [74]	RCT	Athletes	8(NS)	SP increased 2,3-DPG concentration and maximal oxygen uptake increased, compared to placebo.	Moderate
	Buck, *et al.*, 2014 [75]	RCT	Athletes	13(13)	SP had no effect on cycling time trial performance, compared to placebo.	Moderate
	West, *et al.*, 2012 [76]	RCT	Non-athletes	22(10)	SP had no effect on VO_{2peak} performance, compared to placebo.	Moderate

	Author (year)	Study design	Participants		Outcome results of supplementation	EPHPP Global rating
			Group	Sample size (n=F)		
	Duffy and Conlee, 1986 [77]	CT	Non-athletes	11(0)	SP had no effect on VO_{2pea}k or power output, compared to placebo.	Weak
	Galloway, *et al.*, 1996 [78]	CT	Athletes<br>Non-athletes	6(0)<br>6(0)	SP had no effect on VO_2 kinetics, heart rate, pulse rate 2,3-DPG or lactate accumulation, compared to placebo.	Moderate
	Zorbas, *et al.*, 2002 [79]	CT	Athletes<br>Non-athletes	20(0)<br>20(0)	During a period of reduced activity SP supplementation intensified the negative phosphate balance evident during low activity in both athletes and non-athletes.	Moderate
**Zinc**						
	Saeedy, *et al.*, 2016 [80]	RCT	Athletes	32(32)	Zn improved estimated VO_{2peak}, similar to that of HITT training and when combined, Zn and HITT showed further improvements in estimated VO2peak.	Moderate
	Davison, *et al.*, 2016 [81]	RCCT	Non-athletes	8(0)	Zn had no effect on VO_{2Peak} or lactate accumulation, but was sufficient to reduce exercise-induced gut permeability.	Moderate
	Khaled, *et al.*, 1999 [82]	RCT	Non-athletes	10(0)	Zn improved estimated VO_{2peak}, with no significant change in the placebo group.	Moderate
	Marques, *et al.*, 2011 [83]	Interpreted time series	Elite athletes	7(0)	In athletes with Zn deficiency, Zn supplementation improved Zn status and following cessation of Zn and replacement with placebo for a subsequent 30 days, Zn concentrations improved further. Zn increased Zn:C ratio and this was positively correlated with the increase in insulin and HOMA2-IR.	Moderate
	Cinar, *et al.*, 2017 [84]	Cohort	Athletes	30(0)	Zn had no effect on thyroid hormone with or without exhaustive exercise.	Weak
	Cınar, *et al.*, 2017 [85]	Cohort	Athletes	30(0)	Zn had no effect on testosterone with or without exhaustive exercise.	Weak
	Shafiei-Neek, *et al.*, 2011 [86]	CT	Athletes	32(0)	Zn increased free testosterone greater following a bout of exhaustive exercise, compared to placebo.	Moderate
	Crouse, *et al.*, 1984 [87]	RCT	Athletes<br>Non-athletes	21(NS)<br>23(NS)	Zn supplementation increased plasma Zn, fasting plasma high-density-lipoprotein, total cholesterol, low-density-lipoprotein, but triglyceride levels did not change.	Moderate
	Grosshauser, *et al.*, 2006 [88]	Cohort	Athletes	36(18)	Zn supplementation improved markers of immune function in athletes, but had no effect on physical performance.	Moderate
**Sodium**						

	Author (year)	Study design	Participants		Outcome results of supplementation	EPHPP Global rating
			Group	Sample size (n=F)		
	Sanders, *et al.*, 2001 [89]	Interpreted time series	Athletes	6(NS)	Na maintained plasma volume and reduced dehydration, but when fluid intake matched sweat rate had little effect on plasma osmolality.	Moderate
	Sims, *et al.*, 2007 [90]	RCCT	Athletes	13(13)	Na showed a greater exercise tolerance in endurance trained female athletes exercising in 32°C heat.	Moderate
	Sims, *et al.*, 2007 [91]	RCCT	Athletes	8(0)	Na showed a greater exercise tolerance in endurance trained male athletes exercising in 32°C heat.	Moderate
	Speedy, *et al.*, 2002 [92]	CT	Athletes	76(NS)	Na had a smaller percent body weight loss than compared to controls and there was no difference in plasma volume between groups.	Moderate
	Zorbas, *et al.*, 2002 [93]	CT	Athletes	30(0)	Na increased body mass, body fat and maintained VO_{2peak}, compared to placebo.	Moderate
	Zorbas, *et al.*, 2002 [94]	CT	Athletes	30(0)	Na maintained VO_{2peak} during a period of bed rest.	Moderate
	Anastasiou, *et al.*, 2009 [95]	RCCT	Athletes	13(0)	Na maintained plasma osmolality with no change in plasma volume.	Moderate
	Barr, *et al.*, 1991 [96]	CT	Athletes	8(3)	Na was not superior to water at improving plasma sodium.	Moderate
	Hew-Butler, *et al.*, 2006 [97]	CT	Athletes	145(NS)	There were no differences between Na, placebo, and no supplement groups finishing time, serum sodium concentration, mass, rectal temperature, systolic and diastolic blood pressure during a marathon.	Moderate
	Hamouti, *et al.*, 2011 [98]	Cross-sectional	Athletes<br>Non-athletes	10(NS)<br>10(NS)	There was no difference in Na secretion or Na retention with Na supplementation.	Moderate
	Earhart, *et al.*, 2015 [99]	RCT	Athletes	11(7)	Na, given in increments, had no effect on thermoregulation, endurance exercise sweat rate, perceived heat stress, or skin temperature.	Moderate
	Aschenbach, *et al.*, 2000 [100]	Interpreted time series	Athletes	8(0)	Arm crank ergonometric peak power in Na supplemented college wrestlers was no different compared placebo.	Moderate
	Cosgrove and Black, 2013 [101]	RCTDriller, *et al.*, 2012 [102]	Athletes	9(4)	Na had no effect on endurance performance during a cycling time-trial.	Moderate
	Driller, Williams, Bellinger, Howe, & Fell, 2012)	RCT	Athletes	8(0)	Na did not improve peak cycling power performance compared to placebo.	Moderate
	Zorbas, *et al.*, 1995 [103]	CT	Athletes	30(0)	Na minimized the fluid and electrolyte losses caused by prolonged restriction of muscular activity.	Moderate
**Selenium**						

	Author (year)	Study design	Participants		Outcome results of supplementation	EPHPP Global rating
			Group	Sample size (n=F)		
	Savory, *et al.*, 2012 [104]	RCCT	Non-athletes	20(NS)	Se mitigated the exercise-induced markers of oxidative stress in overweight, compared to placebo.	Moderate
	Shafiei-Neek *et al.*, 2011 [86]	CT	Athletes	32(0)	Se had no additional benefit to exercise-induced increase in testosterone or lactate accumulation.	Moderate
	Margaritis, *et al.*, 1997 [105]	RCT	Non-athletes	24(0)	Se had no effect on mitochondrial activity, myosin heavy chain expression or aerobic performance.	Moderate
	Tessier, *et al.*, 1995 [106]	RCT	Non-athletes	24(0)	Se increased glutathione peroxidase greater than placebo, in response to exercise.	Moderate
	Zamora, *et al.*, 1995 [107]	RCT	Non-athletes	24(0)	Se dampened the rate of exercise-induced mitochondrial density and overall biogenesis.	Moderate
**Chromium**						
	Edwards, *et al.*, 2012 [108]	RCT	Athletes	20(20)	Cr had no effect on fat free mass and fat mass percentage greater after 12/13 weeks but at 26 weeks fat free mass increased and fat mass percentage decreased.	Moderate
	Kaats, *et al.*, 1996 [109]	RCT	Non-athletes	233(175)	Cr reduced percentage body fat and tended to increase fat free mass.	Moderate
	Campbell, *et al.*, 2002 [110]	RCT	Non-athletes	17(17)	Cr had no influence on maximal strength, fat mass or muscle fibre characteristics, compared to placebo.	Moderate
	Campbell, *et al.*, 1999 [111]	RCT	Non-athletes	23(0)	Cr showed no influence over maximal strength or power, fat mass or muscle fibre characteristics, compared to placebo.	Moderate
	Clancy, *et al.*, 1994 [112]	RCT	Athletes	36(0)	Cr showed no influence over maximal strength, fat mass or muscle mass, compared to placebo.	Moderate
	Hallmark, *et al.*, 1996 [113]	RCT	Non-athletes	15(0)	Cr showed no influence over maximal strength, fat mass or muscle mass, compared to placebo.	Moderate
	Hasten, *et al.*, 1992 [114]	RCT	Non-athletes	59(22)	Cr showed no influence over maximal strength, fat mass or muscle mass, compared to placebo.	Moderate
	Livolsi, *et al.*, 2001 [115]	RCT	Athletes	15(15)	Cr showed no influence over maximal strength, fat mass or muscle mass, compared to placebo.	Moderate
	Lukaski, *et al.*, 1996 [116]	CT	Non-athletes	36(0)	Cr, increased transferrin saturation decreased, urinary magnesium excretion, in response to resistance training.	Moderate
	Walker, *et al.*, 1998 [117]	RCT	Athletes	20(20)	Cr showed no influence over maximal strength, fat mass, muscle mass or aerobic power, compared to placebo.	Weak
	Volek, *et al.*, 2006 [118]	RCT	Non-athletes	16(0)	Cr had no effect on glucose, glycogen, glycogen synthesis, glycogen synthase activity, muscle phosphatidylinositol 3-kinase or insulin. During exercise recovery, lactate response was higher in Cr compared to placebo.	Weak

	Author (year)	Study design	Participants		Outcome results of supplementation	EPHPP Global rating
			Group	Sample size (n=F)		
	Lefavi, *et al.*, 1993 [119]	RCT	Athletes	34(0)	Cr had no effect on insulin, glucose, total cholesterol, triglyceride, HDL-cholesterol, TC:HDL, LDL-cholesterol, and one hr post-challenge insulin and glucose.	Moderate
**Boron**						
	Meacham, *et al.*, 1994 [120]	CT	Athletes<br>Non-athletes	17(17)<br>11(11)	Br supplementation resulted in lower phosphorus and magnesium, but was attenuated with physical activity. Br had no effect on bone mineral density (BMD).	Weak
	Meacham, *et al.*, 1995 [121]	CT	Athletes<br>Non-athletes	17(17)<br>11(11)	Br supplementation resulted in lower phosphorus with increased Mg, but was attenuated with physical activity.	Weak
	Ferrando and Green, 1993 [122]	RCT	Athletes	19(0)	Br supplementation had no effect on testosterone, lean body mass, and muscular strength.	Weak
	Volpe-Snyder, *et al.*, 1993 [123]	CT	Athletes<br>Non-athletes	17(17)<br>11(11)	Br supplementation had no effect on BMD or hormonal status.	Weak
	Green and Ferrando, 1994 [124]	RCT	Athletes	19(0)	Br supplementation testosterone, lean body mass, and muscular strength.	Moderate
**Multi Mineral**						
	Del Coso, *et al.*, 2016 [125]	RCT	Athletes	26(NS)	Multi-mineral improved cycle, running performance and finishing time, but not jump performance or isometric strength, compared to placebo.	Moderate
	Shafiei-Neek *et al.,* 2011 [86]	CT	Athletes	32(0)	Combination of Zn-Se had no effect on blood lactate or testosterone.	Moderate
	Barry, *et al.*, 2011 [126]	CT	Athletes	20(0)	Multi-mineral supplementation before exercise attenuated the increase in PTH. There were no effects of Ca on changes in CTX, BAP, and iCa.	Moderate
	Shea, *et al.*, 2014 [127]	RCT	Non-athletes	23(23)	When supplementation started 60min before exercise, iCa decreased in the control condition, but not with multi mineral supplementation. PTH increased after exercise but was attenuated by supplementation. CTX increased only in the controls. When supplementation started 15min before exercise, the exercise-induced decrease in iCa was attenuated but there were no differences in PTH and CTX in the supplemented compared to control groups.	Moderate
	Sherk, *et al.*, 2017 [128]	RCT	Athletes	51(0)	iCa decreased from before to after exercise; the decrease was greater with placebo.	Moderate

RCT, randomised control trial; CT, control trial; CCT, crossover control trial; RCCT, randomised crossover controlled trial; HBmass, haemoglobin mass; iPTH, ionised parathyroid hormone; HOMA2-IR, Homeostatic Model Assessment Index.

References

1. Dressendorfer, R.H.; Keen, C.L.; Wade, C.E.; Claybaugh, J.R.; Timmis, G.C. Development of runner's anemia during a 20-day road race: effect of iron supplements. *International Journal of Sports MedicineI* **1991**, *12*, 332-336, doi:10.1055/s-2007-1024692.
2. Govus, A.D.; Garvican-Lewis, L.A.; Abbiss, C.R.; Peeling, P.; Gore, C.J. Pre-altitude serum ferritin levels and daily oral iron supplement dose mediate iron parameter and hemoglobin mass responses to altitude exposure. *PloS one* **2015**, *10*, e0135120, doi:10.1371/journal.pone.0135120.
3. Villanueva, J.; Soria, M.; Gonzalez-Haro, C.; Ezquerra, L.; Nieto, J.L.; Escanero, J.F. Oral iron treatment has a positive effect on iron metabolism in elite soccer players. *Biological trace element research* **2011**, *142*, 398-406, doi:10.1007/s12011-010-8813-z.
4. Flynn, M.G.; Mackinnon, L.; Gedge, V.; Fahlman, M.; Brickman, T. Influence of iron status and iron supplements on natural killer cell activity in trained women runners. *International journal of sports medicine* **2003**, *24*, 217-222, doi:10.1055/s-2003-39095.
5. Friedmann, B.; Jost, J.; Rating, T.; Weller, E.; Werle, E.; Eckardt, K.U.; Bartsch, P.; Mairbaurl, H. Effects of iron supplementation on total body hemoglobin during endurance training at moderate altitude. *International journal of sports medicine* **1999**, *20*, 78-85, doi:10.1055/s-2007-971097.
6. Fogelholm, M.; Jaakkola, L.; Lampisjarvi, T. Effects of iron supplementation in female athletes with low serum ferritin concentration. *International journal of sports medicine* **1992**, *13*, 158-162, doi:10.1055/s-2007-1021248.
7. Klingshirn, L.A.; Pate, R.R.; Bourque, S.P.; Davis, J.M.; Sargent, R.G. Effect of iron supplementation on endurance capacity in iron-depleted female runners. *Medicine and science in sports and exercise* **1992**, *24*, 819-824.
8. Hegenauer, J.; Strause, L.; Saltman, P.; Dann, D.; White, J.; Green, R. Transitory hematologic effects of moderate exercise are not influenced by iron supplementation. *European Journal of Applied Physiology and Occupational Physiology* **1983**, *52*, 57-61.
9. Jensen, C.A.; Weaver, C.M.; Sedlock, D.A. Iron supplementation and iron status in exercising young women. *The Journal of Nutritional Biochemistry* **1991**, *2*, 368-373.
10. Hinton, P.S.; Sinclair, L.M. Iron supplementation maintains ventilatory threshold and improves energetic efficiency in iron-deficient nonanemic athletes. *European journal of clinical nutrition* **2007**, *61*, 30-39, doi:10.1038/sj.ejcn.1602479.
11. Magazanik, A.; Weinstein, Y.; Abarbanel, J.; Lewinski, U.; Shapiro, Y.; Inbar, O.; Epstein, S. Effect of an iron supplement on body iron status and aerobic capacity of young training women. *European Journal of Applied Physiology and Occupational Physiology* **1991**, *62*, 317-323.
12. LaManca, J.J.; Haymes, E.M. Effects of iron repletion on VO2max, endurance, and blood lactate in women. *Medicine and science in sports and exercise* **1993**, *25*, 1386-1392.
13. Hinton, P.S.; Giordano, C.; Brownlie, T.; Haas, J.D. Iron supplementation improves endurance after training in iron-depleted, nonanemic women. *J Appl Physiol* **2000**, *88*, 1103-1111, doi:10.1152/jappl.2000.88.3.1103.
14. McClung, J.P.; Karl, J.P.; Cable, S.J.; Williams, K.W.; Nindl, B.C.; Young, A.J.; Lieberman, H.R. Randomized, double-blind, placebo-controlled trial of iron supplementation in female soldiers during military training: effects on iron status, physical performance, and mood. *The American Journal of Cinical Nutrition* **2009**, *90*, 124-131, doi:10.3945/ajcn.2009.27774.
15. DellaValle, D.M.; Haas, J.D. Iron supplementation improves energetic efficiency in iron-depleted female rowers. *Medicine and science in sports and exercise* **2014**, *46*, 1204-1215, doi:10.1249/mss.0000000000000208.
16. Yoshida, T.; Udo, M.; Chida, M.; Ichioka, M.; Makiguchi, K. Dietary iron supplement during severe physical training in competitive female distance runners. *Res Sport Med* **1990**, *1*, 279-285.
17. Ohira, Y.; Edgerton, V.R.; Gardner, G.W.; Senewiratne, B.; Barnard, R.J.; Simpson, D.R. Work capacity, heart rate and blood lactate responses to iron treatment. *British journal of haematology* **1979**, *41*, 365-372.

18. Garvican, L.A.; Saunders, P.U.; Cardoso, T.; Macdougall, I.C.; Lobigs, L.M.; Fazakerley, R.; Fallon, K.E.; Anderson, B.; Anson, J.M.; Thompson, K.G., et al. Intravenous iron supplementation in distance runners with low or suboptimal ferritin. *Medicine and science in sports and exercise* **2014**, *46*, 376-385, doi:10.1249/MSS.0b013e3182a53594.
19. Peeling, P.; Blee, T.; Goodman, C.; Dawson, B.; Claydon, G.; Beilby, J.; Prins, A. Effect of iron injections on aerobic-exercise performance of iron-depleted female athletes. *International journal of sport nutrition and exercise metabolism* **2007**, *17*, 221-231.
20. Woods, A.; Garvican-Lewis, L.A.; Saunders, P.U.; Lovell, G.; Hughes, D.; Fazakerley, R.; Anderson, B.; Gore, C.J.; Thompson, K.G. Four weeks of IV iron supplementation reduces perceived fatigue and mood disturbance in distance runners. *PloS one* **2014**, *9*, e108042, doi:10.1371/journal.pone.0108042.
21. Brutsaert, T.D.; Hernandez-Cordero, S.; Rivera, J.; Viola, T.; Hughes, G.; Haas, J.D. Iron supplementation improves progressive fatigue resistance during dynamic knee extensor exercise in iron-depleted, nonanemic women. *The American journal of clinical nutrition* **2003**, *77*, 441-448, doi:10.1093/ajcn/77.2.441.
22. Mielgo-Ayuso, J.; Zourdos, M.C.; Calleja-Gonzalez, J.; Urdampilleta, A.; Ostojic, S. Iron supplementation prevents a decline in iron stores and enhances strength performance in elite female volleyball players during the competitive season. *Applied Physiology, Nutrition and Metabolism* **2015**, *40*, 615-622, doi:10.1139/apnm-2014-0500.
23. Burden, R.J.; Pollock, N.; Whyte, G.P.; Richards, T.; Moore, B.; Busbridge, M.; Srai, S.K.; Otto, J.; Pedlar, C.R. Effect of Intravenous Iron on Aerobic Capacity and Iron Metabolism in Elite Athletes. *Medicine and science in sports and exercise* **2015**, *47*, 1399-1407, doi:10.1249/mss.0000000000000568.
24. Ishibashi, A.; Maeda, N.; Kamei, A.; Goto, K. Iron supplementation during three consecutive days of endurance training augmented hepcidin levels. *Nutrients* **2017**, *9*, 820, doi:10.3390/nu9080820.
25. Blee, T.; Goodman, C.; Dawson, B.; Stapff, A. The effect of intramuscular iron injections on serum ferritin levels and physical performance in elite netballers. *Journal of science and medicine in sport* **1999**, *2*, 311-321.
26. Powell, P.D.; Tucker, A. Iron supplementation and running performance in female cross-country runners. *International journal of sports medicine* **1991**, *12*, 462-467, doi:10.1055/s-2007-1024714.
27. Ashenden, M.J.; Pyne, D.B.; Parisotto, R.; Dobson, G.P.; Hahn, A.G. Can reticulocyte parameters be of use in detecting iron deficient erythropoiesis in female athletes? *The Journal of sports medicine and physical fitness* **1999**, *39*, 140-146.
28. Newhouse, I.J.; Clement, D.B.; Taunton, J.E.; McKenzie, D.C. The effects of prelatent/latent iron deficiency on physical work capacity. *Medicine and science in sports and exercise* **1989**, *21*, 263-268.
29. Telford, R.D.; Bunney, C.J.; Catchpole, E.A.; Catchpole, W.R.; Deakin, V.; Gray, B.; Hahn, A.G.; Kerr, D.A. Plasma ferritin concentration and physical work capacity in athletes. *International journal of sport nutrition* **1992**, *2*, 335-342.
30. Martin, B.R.; Davis, S.; Campbell, W.W.; Weaver, C.M. Exercise and calcium supplementation: effects on calcium homeostasis in sportswomen. *Medicine and science in sports and exercise* **2007**, *39*, 1481-1486, doi:10.1249/mss.0b013e318074ccc7.
31. Zorbas, Y.G.; Petrov, K.L.; Kakurin, V.J.; Kuznetsov, N.A.; Charapakhin, K.P.; Alexeyev, I.D.; Denogradov, S.D. Calcium supplementation effect on calcium balance in endurance-trained athletes during prolonged hypokinesia and ambulatory conditions. *Biological trace element research* **2000**, *73*, 231-250, doi:10.1385/bter:73:3:231.
32. Zorbas, Y.G.; Kakurin, V.J.; Kuznetsov, N.A.; Yarullin, V.L.; Andreyev, I.D.; Charapakhin, K.P. Measurements in calcium-supplemented athletes during and after hypokinetic and ambulatory conditions. *Clinical biochemistry* **2000**, *33*, 393-404.
33. Kohrt, W.M.; Wherry, S.J.; Wolfe, P.; Sherk, V.D.; Wellington, T.; Swanson, C.M.; Weaver, C.M.; Boxer, R.S. Maintenance of serum ionized calcium during exercise attenuates parathyroid hormone and bone resorption responses. *Journal of Bone and Mineral Research* **2018**, *33*, 1326-1334, doi:10.1002/jbmr.3428.
34. Guillemant, J.; Accarie, C.; Peres, G.; Guillemant, S. Acute effects of an oral calcium load on markers of bone metabolism during endurance cycling exercise in male athletes. *Calcified tissue international* **2004**, *74*, 407-414, doi:10.1007/s00223-003-0070-0.

35. Haakonssen, E.C.; Ross, M.L.; Knight, E.J.; Cato, L.E.; Nana, A.; Wluka, A.E.; Cicuttini, F.M.; Wang, B.H.; Jenkins, D.G.; Burke, L.M. The effects of a calcium-rich pre-exercise meal on biomarkers of calcium homeostasis in competitive female cyclists: a randomised crossover trial. *PloS one* **2015**, *10*, e0123302, doi:10.1371/journal.pone.0123302.
36. Cinar, V.; Baltaci, A.K.; Mogulkoc, R. Effect of exhausting exercise and calcium supplementation on potassium, magnesium, copper, zinc and calcium levels in athletes. *Pakistan Journal of Medical Sciences* **2009**, *25*, 238-242.
37. Cinar, V.; Baltaci, A.K.; Mogulkoc, R.; Kilic, M. Testosterone levels in athletes at rest and exhaustion: effects of calcium supplementation. *Biological trace element research* **2009**, *129*, 65-69, doi:10.1007/s12011-008-8294-5.
38. Cinar, V.; Cakmakci, O.; Mogulkoc, R.; Baltaci, A.K. Effects of exhaustion and calcium supplementation on adrenocorticotropic hormone and cortisol levels in athletes. *Biological trace element research* **2009**, *127*, 1-5, doi:10.1007/s12011-008-8217-5.
39. Cinar, V.; Mogulkoc, R.; Baltaci, A.K. Calcium supplementation and 4-week exercise on blood parameters of athletes at rest and exhaustion. *Biological trace element research* **2010**, *134*, 130-135, doi:10.1007/s12011-009-8459-x.
40. Cinar, V.; Mogulkoc, R.; Baltaci, A.K.; Bostanci, O. Effects of calcium supplementation on glucose and insulin levels of athletes at rest and after exercise. *Biological trace element research* **2010**, *133*, 29-33, doi:10.1007/s12011-009-8409-7.
41. Cordova Martinez, A.; Fernandez-Lazaro, D.; Mielgo-Ayuso, J.; Seco Calvo, J.; Caballero Garcia, A. Effect of magnesium supplementation on muscular damage markers in basketball players during a full season. *Magnesium research* **2017**, *30*, 61-70, doi:10.1684/mrh.2017.0424.
42. Molina-Lopez, J.; Molina, J.M.; Chirosa, L.J.; Florea, D.; Saez, L.; Millan, E.; Planells, E. Association between erythrocyte concentrations of magnesium and zinc in high-performance handball players after dietary magnesium supplementation. *Magnesium research* **2012**, *25*, 79-88, doi:10.1684/mrh.2012.0311.
43. Setaro, L.; Santos-Silva, P.R.; Nakano, E.Y.; Sales, C.H.; Nunes, N.; Greve, J.M.; Colli, C. Magnesium status and the physical performance of volleyball players: effects of magnesium supplementation. *Journal of sports sciences* **2014**, *32*, 438-445, doi:10.1080/02640414.2013.828847.
44. Brilla, L.R.; Haley, T.F. Effect of magnesium supplementation on strength training in humans. *Journal of the American College of Nutrition* **1992**, *11*, 326-329.
45. Dmitrasinovic, G.; Pesic, V.; Stanic, D.; Plecas-Solarovic, B.; Dajak, M.; Ignjatovic, S. ACTH, cortisol and IL-6 Levels in athletes following magnesium supplementation. *Journal of medical biochemistry* **2016**, *35*, 375-384, doi:10.1515/jomb-2016-0021.
46. Finstad, E.W.; Newhouse, I.J.; Lukaski, H.C.; McAuliffe, J.E.; Stewart, C.R. The effects of magnesium supplementation on exercise performance. *Medicine and science in sports and exercise* **2001**, *33*, 493-498.
47. Kass, L.S.; Skinner, P.; Poeira, F. A pilot study on the effects of magnesium supplementation with high and low habitual dietary magnesium intake on resting and recovery from aerobic and resistance exercise and systolic blood pressure. *Journal of Sports Science and Medicine* **2013**, *12*, 144-150.
48. Zorbas, Y.G.; Kakurin, V.J.; Afonin, V.B.; Charapakhin, K.P.; Denogradov, S.D. Magnesium supplements' effect on magnesium balance in athletes during prolonged restriction of muscular activity. *Kidney and Blood Pressure Research* **1999**, *22*, 146-153, doi:10.1159/000025921.
49. Zorbas, Y.G.; Kakurin, A.G.; Kuznetsov, N.K.; Federov, M.A.; Yaroshenko, Y.Y. Magnesium loading effect on magnesium deficiency in endurance-trained subjects during prolonged restriction of muscular activity. *Biological trace element research* **1998**, *63*, 149-166, doi:10.1007/bf02778874.
50. Porta, S.; Gell, H.; Pichlkastner, K.; Porta, J.; von Ehrlich, B.; Vormann, J.; Stossier, H.; Kisters, K. A system of changes of ionized blood Mg through sports and supplementation. *Tr El Electro* **2013**, *30*, 105-107.
51. Terblanche, S.; Noakes, T.D.; Dennis, S.C.; Marais, D.; Eckert, M. Failure of magnesium supplementation to influence marathon running performance or recovery in magnesium-replete subjects. *International journal of sport nutrition* **1992**, *2*, 154-164.
52. Weller, E.; Bachert, P.; Meinck, H.M.; Friedmann, B.; Bartsch, P.; Mairbaurl, H. Lack of effect of oral Mg-supplementation on Mg in serum, blood cells, and calf muscle. *Medicine and science in sports and exercise* **1998**, *30*, 1584-1591.
53. Petrovic, J.; Stanic, D.; Dmitrasinovic, G.; Plecas-Solarovic, B.; Ignjatovic, S.; Batinic, B.; Popovic, D.; Pesic, V. Magnesium supplementation diminishes peripheral blood lymphocyte DNA oxidative damage in athletes and sedentary young man. *Oxidative medicine and cellular longevity* **2016**, *2016*, 2019643, doi:10.1155/2016/2019643.

54. Cinar, V.; Mogulkoc, R.; Baltaci, A.K.; Nizamlioglu, M. Effect of magnesium supplementation on some plasma elements in athletes at rest and exhaustion. *Biological trace element research* **2007**, *119*, 97-102, doi:10.1007/s12011-007-0024-x.
55. Cinar, V.; Mogulkoc, R.; Baltaci, A.K.; Polat, Y. Adrenocorticotropic hormone and cortisol levels in athletes and sedentary subjects at rest and exhaustion: effects of magnesium supplementation. *Biological trace element research* **2008**, *121*, 215-220, doi:10.1007/s12011-007-8052-0.
56. Cinar, V.; Nizamlioglu, M.; Mogulkoc, R.; Baltaci, A.K. Effects of magnesium supplementation on blood parameters of athletes at rest and after exercise. *Biological trace element research* **2007**, *115*, 205-212, doi:10.1007/bf02685995.
57. Cinar, V.; Polat, Y.; Baltaci, A.K.; Mogulkoc, R. Effects of magnesium supplementation on testosterone levels of athletes and sedentary subjects at rest and after exhaustion. *Biological trace element research* **2011**, *140*, 18-23, doi:10.1007/s12011-010-8676-3.
58. Cinar, V.; Polat, Y.; Mogulkoc, R.; Nizamlioglu, M.; Baltaci, A.K. The effect of magnesium supplementation on glucose and insulin levels of tae-kwan-do sportsmen and sedentary subjects. *Pakistan journal of pharmaceutical sciences* **2008**, *21*, 237-240.
59. Kass, L.S.; Poeira, F. The effect of acute vs chronic magnesium supplementation on exercise and recovery on resistance exercise, blood pressure and total peripheral resistance on normotensive adults. *Journal of the International Society of Sports Nutrition* **2015**, *12*, 19, doi:10.1186/s12970-015-0081-z.
60. Veronese, N.; Berton, L.; Carraro, S.; Bolzetta, F.; De Rui, M.; Perissinotto, E.; Toffanello, E.D.; Bano, G.; Pizzato, S.; Miotto, F., et al. Effect of oral magnesium supplementation on physical performance in healthy elderly women involved in a weekly exercise program: a randomized controlled trial. *The American journal of clinical nutrition* **2014**, *100*, 974-981, doi:10.3945/ajcn.113.080168.
61. Gulick, D.T.; Agarwal, M.; Josephs, J.; Reinmiller, A.; Zimmerman, B. Effects of MagPro™ on muscle performance. *The Journal of Strength and Conditioning Research* **2012**, *26*, 2478-2483.
62. Mooren, F.C.; Golf, S.W.; Volker, K. Effect of magnesium on granulocyte function and on the exercise induced inflammatory response. *Magnesium research* **2003**, *16*, 49-58.
63. Buck, C.; Guelfi, K.; Dawson, B.; McNaughton, L.; Wallman, K. Effects of sodium phosphate and caffeine loading on repeated-sprint ability. *Journal of sports sciences* **2015**, *33*, 1971-1979, doi:10.1080/02640414.2015.1025235.
64. Buck, C.L.; Henry, T.; Guelfi, K.; Dawson, B.; McNaughton, L.R.; Wallman, K. Effects of sodium phosphate and beetroot juice supplementation on repeated-sprint ability in females. *European journal of applied physiology* **2015**, *115*, 2205-2213, doi:10.1007/s00421-015-3201-1.
65. Kopec, B.J.; Dawson, B.T.; Buck, C.; Wallman, K.E. Effects of sodium phosphate and caffeine ingestion on repeated-sprint ability in male athletes. *Journal of science and medicine in sport* **2016**, *19*, 272-276, doi:10.1016/j.jsams.2015.04.001.
66. Brewer, C.P.; Dawson, B.; Wallman, K.E.; Guelfi, K.J. Effect of sodium phosphate supplementation on repeated high-intensity cycling efforts. *Journal of sports sciences* **2015**, *33*, 1109-1116, doi:10.1080/02640414.2014.989536.
67. Folland, J.P.; Stern, R.; Brickley, G. Sodium phosphate loading improves laboratory cycling time-trial performance in trained cyclists. *Journal of science and medicine in sport* **2008**, *11*, 464-468, doi:10.1016/j.jsams.2007.04.004.
68. Brewer, C.P.; Dawson, B.; Wallman, K.E.; Guelfi, K.J. Effect of repeated sodium phosphate loading on cycling time-trial performance and VO2peak. *International journal of sport nutrition and exercise metabolism* **2013**, *23*, 187-194.
69. Goss, F.; Robertson, R.; Riechman, S.; Zoeller, R.; Dabayebeh, I.D.; Moyna, N.; Boer, N.; Peoples, J.; Metz, K. Effect of potassium phosphate supplementation on perceptual and physiological responses to maximal graded exercise. *International journal of sport nutrition and exercise metabolism* **2001**, *11*, 53-62.
70. Kreider, R.B.; Miller, G.W.; Schenck, D.; Cortes, C.W.; Miriel, V.; Somma, C.T.; Rowland, P.; Turner, C.; Hill, D. Effects of phosphate loading on metabolic and myocardial responses to maximal and endurance exercise. *International journal of sport nutrition* **1992**, *2*, 20-47.
71. Czuba, M.; Zajac, A.; Poprzecki, S.; Cholewa, J.; Woska, S. Effects of sodium phosphate loading on aerobic power and capacity in off road cyclists. *Journal of Sports Science and Medicine* **2009**, *8*, 591-599.
72. Czuba, M.; Zając, A.; Poprzecki, S.; Cholewa, J. The influence of sodium phosphate supplementation on VO2max, serum 2, 3-diphosphoglycerate level and heart rate in off-road cyclists. *Journal of Human Kinetics* **2008**, *19*, 149-164.

73. Cade, R.; Conte, M.; Zauner, C.; Mars, D.; Peterson, J.; Lunne, D.; Hommen, N.; Packer, D. Effects of phosphate loading on 2,3-diphosphoglycerate and maximal oxygen uptake. *Medicine and science in sports and exercise* **1984**, *16*, 263-268.
74. Stewart, I.; McNaughton, L.; Davies, P.; Tristram, S. Phosphate loading and the effects on VO2max in trained cyclists. *Research quarterly for exercise and sport* **1990**, *61*, 80-84, doi:10.1080/02701367.1990.10607481.
75. Buck, C.L.; Dawson, B.; Guelfi, K.J.; McNaughton, L.; Wallman, K.E. Sodium phosphate supplementation and time trial performance in female cyclists. *Journal of Sports Science and Medicine* **2014**, *13*, 469-475.
76. West, J.S.; Ayton, T.; Wallman, K.E.; Guelfi, K.J. The effect of 6 days of sodium phosphate supplementation on appetite, energy intake, and aerobic capacity in trained men and women. *International journal of sport nutrition and exercise metabolism* **2012**, *22*, 422-429.
77. Duffy, D.J.; Conlee, R.K. Effects of phosphate loading on leg power and high intensity treadmill exercise. *Medicine and science in sports and exercise* **1986**, *18*, 674-677.
78. Galloway, S.D.; Tremblay, M.S.; Sexsmith, J.R.; Roberts, C.J. The effects of acute phosphate supplementation in subjects of different aerobic fitness levels. *European Journal of Applied Physiology and Occupational Physiology* **1996**, *72*, 224-230.
79. Zorbas, Y.G.; Kakurin, V.J.; Kuznetsov, N.A.; Yarullin, V.L.; Andreyev, I.D.; Charapakhin, K.P. Phosphate deposition capacity of athletes during hypokinesia, phosphate loading, and ambulation. *Biological trace element research* **2002**, *85*, 211-226, doi:10.1385/bter:85:3:211.
80. Saeedy, M.; Bijeh, N.; Moazzami, M. The effect of six weeks of high-intensity interval training with and without zinc supplementation on aerobic power and anaerobic power in female futsal players. *International Journal of Applied Exercise Physiology* **2016**, *5*, 1-10.
81. Davison, G.; Marchbank, T.; March, D.S.; Thatcher, R.; Playford, R.J. Zinc carnosine works with bovine colostrum in truncating heavy exercise-induced increase in gut permeability in healthy volunteers. *The American journal of clinical nutrition* **2016**, *104*, 526-536, doi:10.3945/ajcn.116.134403.
82. Khaled, S.; Brun, J.F.; Cassanas, G.; Bardet, L.; Orsetti, A. Effects of zinc supplementation on blood rheology during exercise. *Clinical hemorheology and microcirculation* **1999**, *20*, 1-10.
83. Marques, L.F.; Donangelo, C.M.; Franco, J.G.; Pires, L.; Luna, A.S.; Casimiro-Lopes, G.; Lisboa, P.C.; Koury, J.C. Plasma zinc, copper, and serum thyroid hormones and insulin levels after zinc supplementation followed by placebo in competitive athletes. *Biological trace element research* **2011**, *142*, 415-423, doi:10.1007/s12011-010-8821-z.
84. Cinar, V.; Akbulut, T.; Sarikaya, M. Effect of zinc supplement and weight lifting exercise on thyroid hormone levels. *Indian Journal of Physiology and Pharmacology* **2017**, *61*, 232-236.
85. Cınar, V.; Talaghir, L.G.; Akbulut, T.; Turgut, M.; Sarıkaya, M. The effects of the zinc supplementation and weight trainings on the testosterone levels. *Hum Sport Med* **2017**, *17*, 58-63.
86. Shafiei-Neek, L.; Gaeini, A.A.; Choobineh, S. Effect of zinc and selenium supplementation on serum testosterone and plasma lactate in cyclist after an exhaustive exercise bout. *Biological trace element research* **2011**, *144*, 454-462, doi:10.1007/s12011-011-9138-2.
87. Crouse, S.F.; Hooper, P.L.; Atterbom, H.A.; Papenfuss, R.L. Zinc ingestion and lipoprotein values in sedentary and endurance-trained men. *Journal of the American Medical Association* **1984**, *252*, 785-787.
88. Grosshauser, M.; Becker, K.; Riemann, D.; Langner, J.; Stangi, G.; Eder, K. The effect of daily zinc supplementation on immune system of athletes. *Tr El Electro* **2006**, *23*.
89. Sanders, B.; Noakes, T.D.; Dennis, S.C. Sodium replacement and fluid shifts during prolonged exercise in humans. *European journal of applied physiology* **2001**, *84*, 419-425, doi:10.1007/s004210000371.
90. Sims, S.T.; Rehrer, N.J.; Bell, M.L.; Cotter, J.D. Preexercise sodium loading aids fluid balance and endurance for women exercising in the heat. *J Appl Physiol* **2007**, *103*, 534-541, doi:10.1152/japplphysiol.01203.2006.
91. Sims, S.T.; van Vliet, L.; Cotter, J.D.; Rehrer, N.J. Sodium loading aids fluid balance and reduces physiological strain of trained men exercising in the heat. *Medicine and science in sports and exercise* **2007**, *39*, 123-130, doi:10.1249/01.mss.0000241639.97972.4a.

92. Speedy, D.B.; Thompson, J.M.; Rodgers, I.; Collins, M.; Sharwood, K.; Noakes, T.D. Oral salt supplementation during ultradistance exercise. *Clinical Journal of Sport Medicine* **2002**, *12*, 279-284.
93. Zorbas, Y.G.; Kakurin, V.J.; Kuznetsov, N.A.; Yarullin, V.L. Fluid and salt supplementation effect on body hydration and electrolyte homeostasis during bed rest and ambulation. *Acta astronautica* **2002**, *50*, 765-774.
94. Zorbas, Y.G.; Petrov, K.L.; Yarullin, V.L.; Kakurin, V.J.; Popov, V.K.; Deogeneov, V.A. Effect of fluid and salt supplementation on body hydration of athletes during prolonged hypokinesia. *Acta astronautica* **2002**, *50*, 641-651.
95. Anastasiou, C.A.; Kavouras, S.A.; Arnaoutis, G.; Gioxari, A.; Kollia, M.; Botoula, E.; Sidossis, L.S. Sodium replacement and plasma sodium drop during exercise in the heat when fluid intake matches fluid loss. *Journal of Athletic Training* **2009**, *44*, 117-123.
96. Barr, S.I.; Costill, D.L.; Fink, W.J. Fluid replacement during prolonged exercise: effects of water, saline, or no fluid. *Medicine and science in sports and exercise* **1991**, *23*, 811-817.
97. Hew-Butler, T.D.; Sharwood, K.; Collins, M.; Speedy, D.; Noakes, T. Sodium supplementation is not required to maintain serum sodium concentrations during an Ironman triathlon. *British journal of sports medicine* **2006**, *40*, 255-259, doi:10.1136/bjsm.2005.022418.
98. Hamouti, N.; Del Coso, J.; Ortega, J.F.; Mora-Rodriguez, R. Sweat sodium concentration during exercise in the heat in aerobically trained and untrained humans. *European journal of applied physiology* **2011**, *111*, 2873-2881, doi:10.1007/s00421-011-1911-6.
99. Earhart, E.L.; Weiss, E.P.; Rahman, R.; Kelly, P.V. Effects of oral sodium supplementation on indices of thermoregulation in trained, endurance athletes. *Journal of Sports Science and Medicine* **2015**, *14*, 172-178.
100. Aschenbach, W.; Ocel, J.; Craft, L.; Ward, C.; Spangenburg, E.; Williams, J. Effect of oral sodium loading on high-intensity arm ergometry in college wrestlers. *Medicine and science in sports and exercise* **2000**, *32*, 669-675.
101. Cosgrove, S.D.; Black, K.E. Sodium supplementation has no effect on endurance performance during a cycling time-trial in cool conditions: a randomised cross-over trial. *Journal of the International Society of Sports Nutrition* **2013**, *10*, 30, doi:10.1186/1550-2783-10-30.
102. Driller, M.; Williams, A.; Bellinger, P.; Howe, S.; Fell, J. The effects of NaHCO3 and NaCl loading on hematocrit and high-intensity cycling performance. *Journal of Exercise Physiology* **2012**, *15*, 47-57.
103. Zorbas, Y.G.; Federenko, Y.F.; Naexu, K.A. Effect of daily hyperhydration on fluid-electrolyte changes in endurance-trained volunteers during prolonged restriction of muscular activity. *Biological trace element research* **1995**, *50*, 57-78, doi:10.1007/bf02789149.
104. Savory, L.A.; Kerr, C.J.; Whiting, P.; Finer, N.; McEneny, J.; Ashton, T. Selenium supplementation and exercise: effect on oxidant stress in overweight adults. *Obesity* **2012**, *20*, 794-801, doi:10.1038/oby.2011.83.
105. Margaritis, I.; Tessier, F.; Prou, E.; Marconnet, P.; Marini, J.F. Effects of endurance training on skeletal muscle oxidative capacities with and without selenium supplementation. *Journal of Trace Elements in Medicine and Biology* **1997**, *11*, 37-43, doi:10.1016/s0946-672x(97)80008-9.
106. Tessier, F.; Margaritis, I.; Richard, M.J.; Moynot, C.; Marconnet, P. Selenium and training effects on the glutathione system and aerobic performance. *Medicine and science in sports and exercise* **1995**, *27*, 390-396.
107. Zamora, A.J.; Tessier, F.; Marconnet, P.; Margaritis, I.; Marini, J.F. Mitochondria changes in human muscle after prolonged exercise, endurance training and selenium supplementation. *European Journal of Applied Physiology and Occupational Physiology* **1995**, *71*, 505-511.
108. Edwards, W.; Pringle, D.; Palfrey, T.; Anderson, D. Effects of chromium picolinate supplementation on body composition in in-season division I intercollegiate female swimmers. *Medicina Sportiva* **2012**, *16*, 99-103.
109. Kaats, G.R.; Blum, K.; Fisher, J.A.; Adelman, J.A. Effects of chromium picolinate supplementation on body composition: a randomized, double-masked, placebo-controlled study. *Current Therapeutic Research* **1996**, *57*, 747-756.
110. Campbell, W.W.; Joseph, L.J.; Anderson, R.A.; Davey, S.L.; Hinton, J.; Evans, W.J. Effects of resistive training and chromium picolinate on body composition and skeletal muscle size in older women. *International journal of sport nutrition and exercise metabolism* **2002**, *12*, 125-135.
111. Campbell, W.W.; Joseph, L.J.; Davey, S.L.; Cyr-Campbell, D.; Anderson, R.A.; Evans, W.J. Effects of resistance training and chromium picolinate on body composition and skeletal muscle in older men. *J Appl Physiol* **1999**, *86*, 29-39, doi:10.1152/jappl.1999.86.1.29.

112. Clancy, S.P.; Clarkson, P.M.; DeCheke, M.E.; Nosaka, K.; Freedson, P.S.; Cunningham, J.J.; Valentine, B. Effects of chromium picolinate supplementation on body composition, strength, and urinary chromium loss in football players. *International journal of sport nutrition* **1994**, *4*, 142-153.
113. Hallmark, M.A.; Reynolds, T.H.; DeSouza, C.A.; Dotson, C.O.; Anderson, R.A.; Rogers, M.A. Effects of chromium and resistive training on muscle strength and body composition. *Medicine and science in sports and exercise* **1996**, *28*, 139-144.
114. Hasten, D.L.; Rome, E.P.; Franks, B.D.; Hegsted, M. Effects of chromium picolinate on beginning weight training students. *International journal of sport nutrition* **1992**, *2*, 343-350.
115. Livolsi, J.M.; Adams, G.M.; Laguna, P.L. The effect of chromium picolinate on muscular strength and body composition in women athletes. *Journal of strength and conditioning research* **2001**, *15*, 161-166.
116. Lukaski, H.C.; Bolonchuk, W.W.; Siders, W.A.; Milne, D.B. Chromium supplementation and resistance training: effects on body composition, strength, and trace element status of men. *The American journal of clinical nutrition* **1996**, *63*, 954-965, doi:10.1093/ajcn/63.6.954.
117. Walker, L.S.; Bemben, M.G.; Bemben, D.A.; Knehans, A.W. Chromium picolinate effects on body composition and muscular performance in wrestlers. *Medicine and science in sports and exercise* **1998**, *30*, 1730-1737.
118. Volek, J.S.; Silvestre, R.; Kirwan, J.P.; Sharman, M.J.; Judelson, D.A.; Spiering, B.A.; Vingren, J.L.; Maresh, C.M.; Vanheest, J.L.; Kraemer, W.J. Effects of chromium supplementation on glycogen synthesis after high-intensity exercise. *Medicine and science in sports and exercise* **2006**, *38*, 2102-2109, doi:10.1249/01.mss.0000235353.09061.54.
119. Lefavi, R.G.; Wilson, G.D.; Keith, R.E.; Anderson, R.A.; Blessing, D.L.; Hames, C.G.; McMillan, J.L. Lipid-lowering effect of a dietary chromium (III)—Nicotinic acid complex in male athletes. *Nutrition Research* **1993**, *13*, 239-249.
120. Meacham, S.L.; Taper, L.J.; Volpe, S.L. Effects of boron supplementation on bone mineral density and dietary, blood, and urinary calcium, phosphorus, magnesium, and boron in female athletes. *Environmental health perspectives* **1994**, *102* 79-82.
121. Meacham, S.L.; Taper, L.J.; Volpe, S.L. Effect of boron supplementation on blood and urinary calcium, magnesium, and phosphorus, and urinary boron in athletic and sedentary women. *The American journal of clinical nutrition* **1995**, *61*, 341-345, doi:10.1093/ajcn/61.2.341.
122. Ferrando, A.A.; Green, N.R. The effect of boron supplementation on lean body mass, plasma testosterone levels, and strength in male bodybuilders. *International journal of sport nutrition* **1993**, *3*, 140-149.
123. Volpe-Snyder, S.L.; Taper, L.J.; Meacham, S.L. The effect of boron supplementation on bone mineral density and hormonal status in college female athletes. *Med Exerc Nutr Health* **1993**, *2*, 323-330.
124. Green, N.R.; Ferrando, A.A. Plasma boron and the effects of boron supplementation in males. *Environmental health perspectives* **1994**, *102*, 73-77.
125. Del Coso, J.; Gonzalez-Millan, C.; Salinero, J.J.; Abian-Vicen, J.; Areces, F.; Lledo, M.; Lara, B.; Gallo-Salazar, C.; Ruiz-Vicente, D. Effects of oral salt supplementation on physical performance during a half-ironman: A randomized controlled trial. *Scandinavian Journal of Medicine and Science in Sports* **2016**, *26*, 156-164, doi:10.1111/sms.12427.
126. Barry, D.W.; Hansen, K.C.; van Pelt, R.E.; Witten, M.; Wolfe, P.; Kohrt, W.M. Acute calcium ingestion attenuates exercise-induced disruption of calcium homeostasis. *Medicine and science in sports and exercise* **2011**, *43*, 617-623, doi:10.1249/MSS.0b013e3181f79fa8.
127. Shea, K.L.; Barry, D.W.; Sherk, V.D.; Hansen, K.C.; Wolfe, P.; Kohrt, W.M. Calcium supplementation and parathyroid hormone response to vigorous walking in postmenopausal women. *Medicine and science in sports and exercise* **2014**, *46*, 2007-2013, doi:10.1249/mss.0000000000000320.
128. Sherk, V.D.; Wherry, S.J.; Barry, D.W.; Shea, K.L.; Wolfe, P.; Kohrt, W.M. Calcium supplementation attenuates disruptions in calcium homeostasis during exercise. *Medicine and science in sports and exercise* **2017**, *49*, 1437-1442, doi:10.1249/mss.0000000000001239.
